# Midlife body mass index, central adiposity and neuropsychological performance over 10 years in women living with and without HIV

**DOI:** 10.3389/fendo.2023.1108313

**Published:** 2023-07-07

**Authors:** Elizabeth Vásquez, Mark H. Kuniholm, Allison A. Appleton, Leah H. Rubin, Ada A. Adimora, Margaret A. Fischl, Ervin Fox, Wendy J. Mack, Susan Holman, Caitlin Anne Moran, Howard Minkoff, Michael W. Plankey, Anjali Sharma, Phyllis C. Tien, Kathleen M. Weber, Deborah R. Gustafson

**Affiliations:** ^1^ Department of Epidemiology and Biostatistics, University at Albany School of Public Health State University of New York, Rensselaer, NY, United States; ^2^ Department of Neurology, Johns Hopkins University, Baltimore, MD, United States; ^3^ Department of Psychology, Johns Hopkins University, Baltimore, MD, United States; ^4^ Department Epidemiology, Johns Hopkins University, Baltimore, MD, United States; ^5^ Department of Medicine, University of North Carolina at Chapel Hill, Chapel Hill, NC, United States; ^6^ Department of Epidemiology, University of North Carolina at Chapel Hill, Chapel Hill, NC, United States; ^7^ Department of Medicine, University of Miami Miller School of Medicine, Miami, FL, United States; ^8^ Department of Medicine, University of Mississippi Medical Center, Jackson, MS, United States; ^9^ Population and Public Health Sciences, University of Southern California, Los Angeles, CA, United States; ^10^ Department of Medicine/STAR Program, State University of New York Health Sciences University, Brooklyn, NY, United States; ^11^ Department of Medicine, Emory University School of Medicine, Atlanta, GA, United States; ^12^ Grady Healthcare System, Infectious Diseases Program, Atlanta, United States; ^13^ Department of Neurology, State of New York Downstate Health Sciences University, Brooklyn, NY, United States; ^14^ Department of Medicine, Georgetown University, Washington, DC, United States; ^15^ Department of Medicine, Albert Einstein College of Medicine, Bronx, NY, United States; ^16^ Department of Medicine, University of California, San Francisco, CA, United States; ^17^ Department of Veterans Affairs, San Francisco, CA, United States; ^18^ Cook County Health/Hektoen Institute of Medicine, Chicago, IL, United States

**Keywords:** body mass index, neuropsychological performance, HIV, aging, waist circumference, obesity, women

## Abstract

**Background and objective:**

Observations of overweight and obesity in association with neuropsychological performance (NP) vary over the adult life course depending on baseline levels, biological sex, age, race, temporality of measurements, and other factors. Therefore, similar published analyses across cohorts are inconsistent. In our sample of women living with HIV (WLWH) and women without HIV (WWOH), we conducted comparable analyses as those published in men with and without HIV. We examined cross-sectional and longitudinal associations between body mass index (BMI) and waist circumference (WC) and NP.

**Methods:**

Four hundred thirty two 432 virologically-suppressed WLWH and 367 WWOH, ≥40 years in the Women’s Interagency HIV Study (WIHS) with anthropometry and NP assessments every two years from 2009-2019 were included in the study. Demographically-adjusted T-scores were calculated for six NP domains: learning, memory, executive function, processing speed, attention and working memory, and motor function. Multivariable linear regression models stratified by HIV status were used to examine cross-sectional associations of BMI and WC by NP domain; repeated measures analyses assessed baseline BMI and WC in association with longitudinal change in NP. Covariates included sociodemographic, behavioral, and HIV-related characteristics.

**Results:**

At baseline among all women, the median age was 45 years, 65% were Non-Latinx Black women, and 45% were obese women. Obese WLWH (BMI≥30.0 kg/m^2^) had poorer executive function (β=-2.27, 95%CI [-4.46, -0.07]) versus WLWH with healthy BMI (18.5–24.9 kg/m^2^). Longitudinally over ~8 years, obese versus overweight WWOH improved on memory (β=2.19, 95%CI [0.13, 4.26]), however overweight versus healthy WWOH experienced declining memory (β= -2.67, 95%CI [-5.40, -0.07]). Increasing WC was associated with declining executive, processing speed, and motor function (p’s<0.05); an at-risk WC was associated with improved memory (β=1.81, 95%CI [0.19, 3.44]) among WWOH. Among WLWH, increasing BMI was associated with improved learning (β=0.07, 95%CI [0.00, 0.15].

**Conclusion:**

Our cross-sectional and longitudinal analyses evaluating the associations of BMI and WC and NP were mixed compared to previous reports. This illustrates the importance of sociodemographic characteristics, baseline levels of exposures and outcomes, HIV status, temporality of measurements, and other factors when evaluating aging HIV epidemiology study results.

## Introduction

Obesity is a leading cause of morbidity and mortality in the United States (U.S.) and globally (1, 2). Risk factors for obesity among aging adults include a slowing basal metabolic rate, positive energy balance (increased energy intake and/or decreased physical activity), low or worsening dietary quality, and genetics (3). Among women, body weight gain is also due to an increase in adipose versus other body tissues, and can be secondary to diminishing ovarian function and corresponding estrogen levels, and hypothalamic-pituitary-adrenal and fat-brain axis dysregulation (4). Body adiposity is usually clinically measured using anthropometric measures such as body mass index (BMI, kg/m^2^), and central or abdominal adiposity *via* waist circumference (WC). When measured during midlife, some studies suggest that higher levels of either BMI and WC increase risk for subsequent later life cognitive decline, and late-onset Alzheimer’s disease (AD) and Vascular Cognitive Impairments and Dementias (VCID) (5–11).

Adipose tissue is the human body’s largest endocrine organ (12) and secretes more than 700 hormones and proinflammatory cytokines, interleukins, and other bioactive compounds, cumulatively termed adipokines (13, 14). With increasing body adiposity, adipokines contribute to proinflammatory and altered metabolic and vascular risk states, and may cross the blood-brain barrier influencing risk for cognitive impairments, AD and VCID (15). Moreover, the neurovascular unit, where a neuron meets a capillary in the central nervous system (16), is critical for optimal brain function, and may be compromised in obese adults, leading to impaired cognitive function (17).

Central obesity is particularly detrimental for cardiovascular (18) and cerebrovascular disease (19) since it is associated with enhanced deposition of visceral fat, which is more metabolically active than subcutaneous fat (20). In addition, enlargement of the abdominal adipose tissue depot in particular, may exacerbate adipose tissue’s hyper-inflammatory response (21), increase cytokine production and promote other endocrine sequelae (15), influencing brain structure and function – the latter often measured as neuropsychological performance (NP) (15, 22). With the increasing older adult population (23), including among underrepresented and diverse populations, an enhanced understanding of whether higher BMI and central adiposity may lead to impairments and/or changes in neuropsychological performance (NP) in vulnerable populations, such as women living with HIV infection (WLWH) who are surviving to older ages, is needed. Although the number of new HIV cases continues to decrease, women comprise approximate 25% of prevalent (24) and 18% of incident HIV cases in the United States (US) (25) In addition, incident HIV is increasing among women and men older than 50 years of age in the US, and the average age of people with HIV is >50 years in the US when their risk for aging-related NP impairments and decline increase (26).

In an analysis among men living with HIV (MLWH) and without HIV (MWOH) participating in the Multicenter AIDS Cohort Study (MACS), cross-sectionally a higher BMI was inversely associated with motor function and attention/working memory in MWOH only; WC was inversely associated with motor function in MLWH and MWOH (27). Subsequent 10-year NP trajectories analyses showed that an obese BMI was associated with decline in motor performance in MLWH; whereas in MWOH men, obesity was associated with a greater decline in motor function, learning, and memory performance. Our goal was to consider whether similar associations might be observed in WLWH and women without HIV (WWOH) who participated in the Women’s Interagency HIV Study (WIHS) (27). In these analyses, we examined multivariable-adjusted associations of BMI and WC with NP cross-sectionally and prospectively over ~10 years follow-up in virologically-suppressed WLWH and WWOH who were ≥40 years old. Our primary hypothesis was that higher adult BMI and WC would be associated with poorer NP, and this association would be more pronounced among WLWH using both cross-sectional and longitudinal approaches.

## Materials and methods

### Study population

Initiated in 1994, the WIHS was a multisite, prospective cohort of women with or at risk for HIV infection. In 2012, four new clinical research sites in the southern U.S. were added. The WIHS sites included in this analysis were Bronx and Brooklyn, NY; San Francisco, CA; Chicago, IL; Washington, DC; Atlanta, GA; Chapel Hill, NC; Miami, FL; and Birmingham, AL/Jackson, MS. WIHS methods and baseline cohort characteristics have been described (28, 29). The WIHS recruited WLWH and a sociodemographically-similar group of WWOH, and collected survey and clinical data as well as biospecimens in an identical fashion across all clinical research sites. Most women were recruited from clinical settings, *via* postings and word of mouth. Study visits were every six months and independent of clinical care. Frequent contact facilitated high retention of a historically-neglected study population based on race and sociodemographic factors that continues to be under-represented in clinical research in the U.S. Moreover, WIHS data and specimen collection methods were protocol-driven and conducted by dedicated, trained study personnel which allowed for standardized data collection and implementation of routine, integrated quality assurance methods over time.

### Ethical approval and informed consent

The study was approved by the Institutional Review Board (IRB) of each participating site in accordance with the Declaration of Helsinki. Each participating institution’s IRB has been formally designated to review and monitor biomedical research involving human subjects, with the primary responsibility being the protection of subjects from undue risk and from deprivation of personal rights and dignity, which are the cornerstones of ethical research (For the southern sites: University of Mississippi Medical Center; University of North Carolina at Chapel Hill; University of Alabama at Birmingham; University of Miami and Emory University. Brooklyn: SUNY Downstate Medical Center and Kings County Medical Center. Bronx: Montefiore Medical Center; Beth Israel Medical Center; Mount Sinai School of Medicine. Chicago: Cook County Health & Hospitals System; Rush University Medical Center; University of Illinois at Chicago. San Francisco: University of California, San Francisco; Alameda Health System; Sutter Health; Santa Clara Valley Medical Center; San Mateo Medical Center. District of Columbia: Georgetown University; Montgomery County Department of Health and Human Services; Inova; Howard University and Whitman-Walker Clinic. All participants provided written informed consent to undergo clinical exams, blood draws, and all measurements reported herein during each WIHS visit.

### Eligibility criteria

Baseline eligibility criteria for inclusion in this analysis included ≥40 years old, clinically-measured BMI and WC, and no documented dementia. In addition, the full NP battery had to be completed at ≥2 visits. Underweight (<18.5 kg/m^2^) women (N=13) were excluded. WLWH had to be taking ART at baseline and demonstrate an HIV-1 RNA <400 copies/mL at >80% of their NP visits. The HIV-1 RNA <400 copies/mL criterion ensured that only women with consistent ART adherence and virologic suppression were included, and conformed with the methods of a prior analysis that used these thresholds among MLWH participating in the MACS (27).

### Exposure ascertainment: Body mass index and waist circumference

Body weight in kilograms (kg) and body height in meters (m) were clinically measured. BMI was calculated as kg/m^2^. BMI was categorized using traditional guidelines as underweight (<18.5 kg/m^2^), healthy (18.5–24.9 kg/m^2^), overweight (25.0–29.9 kg/m^2^), and obese (≥30 kg/m^2^) (30). Central obesity was clinically measured *via* WC in centimeters (cm) and categorized as high risk (≥88 cm or ≥35 inches) and low risk (<88 cm or <38 inches) (18).

### Outcome ascertainment: Neuropsychological performance assessments

Comprehensive NP assessments were administered in the same order, during one session by a single, certified interviewer at each WIHS clinical research site. The NP assessments included the Rey Auditory Verbal Learning Test (RAVLT), Rey Complex Figure, Trail-Making Test (TMT) Parts A and B, Stroop Color-Interference Test, Symbol Digit Modalities Test (SDMT), California Computerized Assessment Package (CalCAP) Reaction Time Test, and Grooved Pegboard (GPEG). The Wide Range Achievement Test (WRAT) measured intelligence. Demographically-adjusted T-scores for each of six NP domains were calculated as previously reported in the WIHS and MACS (27, 31, 32). Briefly, the assessments used to calculate performance on the six neuropsychological domains were: (1) *Learning* (RAVLT total learning and Rey immediate recall); (2) *Memory* (RAVLT delayed recall and Rey delayed recall); (3) *Executive Function* (TMT-B, Stroop interference trial); (4) *Processing Speed* (SDMT, Stroop color-naming trial); (5) *Attention and Working Memory* (CalCAP mean simple and complex reaction time) and (6) *Motor Function* (GPEG, dominant and nondominant hand) (31, 33). Calculation of T-scores included adjustment for age, education, WRAT score, race (non-Latinx Black people vs non-Latinx White people) and ethnicity (Latinx vs nonLatinx people). WWOH were the reference group. The study visit with completion of the first NP battery was defined as the baseline study visit.

### Covariates

A limited set of covariates was selected based on what has been reported in the literature (27, 31, 34). Covariates included behavioral characteristics such as tobacco use (never, former, and current); weekly alcohol intake using the National Institute on Alcohol Abuse and Alcoholism’s thresholds for heavy alcohol use among women (35) (abstainers [0 drinks per week], moderate [1–7 drinks per week], and excessive [>7 drinks per week]); and use of marijuana, stimulants (nonprescription, including cocaine and methamphetamine), and other illicit drugs, all measured at the NP baseline visit as use versus no use within the previous six months. Clinically-measured covariates included blood pressure with hypertension defined as systolic blood pressure >140 mmHg, diastolic blood pressure >90 mmHg, or self-reported hypertension with use of medications, self-reported diabetes (yes/no), and the presence of clinically-relevant depressive symptoms using the Center for Epidemiological Studies Depression Scale (CES-D) score ≥16. Hepatitis C virus (HCV, uninfected, cleared, or chronic) was also included as a covariate because it is associated with neurological and psychiatric symptoms (36). In analyses of WLWH only, additional covariates included current CD4+ T-cell count, history of AIDS, and use of efavirenz, which is known to influence NP (37–39).

### Data availability

WIHS methods are adequately described herein, and references to previous publications are provided (29, 40, 41). The WIHS provides deidentified data for analytic inquiry on approval by the study investigators (https://statepi.jhsph.edu/mwccs/).

### Statistical analysis

Descriptive statistics were calculated for all variables. BMI and WC were considered as both continuous and categorical exposures. Categories were defined using the cut-offs for overweight, obesity and central obesity. Both continuous and traditional categorical anthropometric measures were used to potentially identify other relevant cut-offs for this sample of women. Univariate associations of BMI and WC with NP at the baseline visit (2009) were analyzed using the Wilcoxon rank sum test and the Fisher’s exact test for continuous and categorical variables, respectively. Multivariable-adjusted general linear regression models were used to examine whether continuous and categorical BMI (healthy, overweight, and obese) and WC (continuous or high vs. low risk) were cross-sectionally associated with NP domains (continuous t-scores) at the participant’s NP baseline visit. Longitudinally, repeated measures of NP regression models, with generalized estimating equations and exchangeable covariance structures adjusting for time-varying and invariant covariates, were used with BMI and WC as predictors. In addition, analyses were conducted within BMI and WC categories. We also evaluated interactions between baseline age and BMI or WC across models and found no significant interactions. All analyses were conducted using SAS (version 9.4, Cary, NC). Results were considered statistically significant at a two-tailed α<0.05 threshold.

## Results

### WIHS participant characteristics at the baseline visit

**Figure 1:**
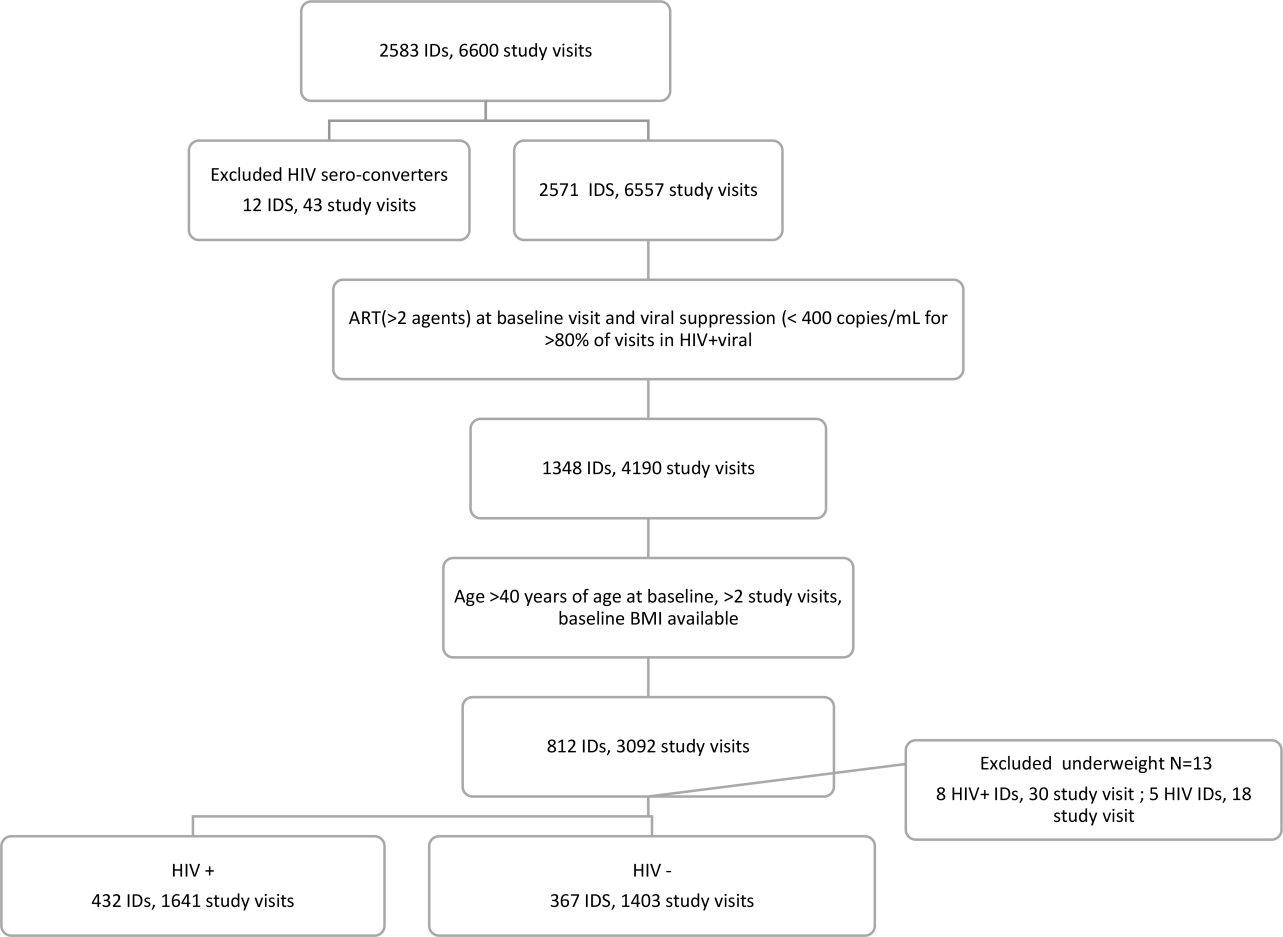
Selection of the analytic sample.

A total of 432 WLWH and 367 WWOH met the inclusion criteria (**Figure 1**). The average length of follow-up was 8.0 ± 1.5 years. Baseline characteristics of the study sample are shown in **Table 1**. At baseline, median age for the total sample was 45 years (age range 41-56) and 65% of women were -non-Latinx Black women. Among WLWH, 28% were overweight and 45% were obese; 69% had a high-risk WC. Obese WLWH were more likely to self-report diabetes (p=0.001) and not have HCV infection (P<0.0001) compared to non-obese WLWH. WLWH participated in a median of 4 (IQR: 3-4) NP visits, totaling 1641 NP study visits. Among WWOH, 26% were overweight and 56% were obese; 75% had a high-risk WC. Obese WWOH were more likely to have hypertension (p=0.003), self-reported diabetes (p=0.005), and higher alcohol (p=0.010) and stimulant drug use (p=0.030) compared to non-obese WWOH. WWOH had a median of 4 (IQR: 3-4) NP visits with a total of 1403 NP study visits.

**Table 1:** Baseline characteristics of the WIHS sample by HIV serostatus and body mass index (BMI) category.

	WWOH (*n*=367)				WLWH (*n*=432)			
	Healthy (BMI 18.5024.99 kg/m^2^) (*n*=64, 17.4%)	Overweight (BMI 25.00–29.99 kg/m^2^) (*n*=96, 26.2%)	Obese (BMI ≥30.00 kg/m^2^) (*n*=207, 56.4%)	P value	Healthy (BMI 18.50–24.99 kg/m^2^) (*n*=115, 26.6%)	Overweight (BMI 25.00–29.99 kg/m^2^) (*n*=122, 28.2%)	Obese (BMI ≥30.00 kg/m^2^) (*n*=195, 45.1%)	*P* value
Age, years, median (IQR)	45 (44, 46)	45 (44, 54)	45 (45, 46)	0.425	45 (44, 45)	45 (44, 46)	46 (45, 46)	0.462
≥ 55 years at baseline, N (%)	7 (11)	11 (11)	31 (15)	0.659	26 (23)	20 (16)	21 (11)	0.021
Calendar year of first visit (Range)	2010 (2009–2010)	2010 (2009–2010)	2010 (2009–2011)	0.603	2010 (2009–2010)	2010 (2009–2010)	2010 (2010–2011)	0.339
Number of visits per participant, median (IQR)	4 (3, 4)	4 (3, 4)	4 (3, 4)	0.186	4 (3, 4)	4 (3, 4)	4 (3, 4)	0.673
Body mass index, kg/m^2^, median (IQR)	22.7 (21.3, 23.8)	28.0 (26.2, 28.9)	36.9 (33.5, 40.7)	<0.0001	22.8 (21.6, 24.0)	27.4 (26.4, 28.5)	35.7 (32.0, 40.4)	<0.0001
Waist circumference, cm, median (IQR)	80.3 (75.9, 84.4)	91.2 (85.8, 96.1)	108.9 (101.9, 119.9)	<0.0001	82.1 (78.4, 85.8)	92.0 (87.8, 97.9)	110.2 (103.3, 120.0)	<0.0001
Race, N (%)				0.644				0.014
Non-Latinx Black	48 (75)	67 (70)	140 (68)		60 (52)	75 (61)	138 (71)	
Non-Latinx White	3 (5)	6 (6)	12 (6)		30 (26)	19 (16)	21 (11)	
Latinx	12 (19)	20 (21)	40 (19)		18 (16)	23 (19)	27 (14)	
Other	1 (2)	3 (3)	15 (7)		7 (6)	5 (4)	9 (5)	
Education N (%)				0.986				0.270
Less than high school	21 (33)	33 (35)	66 (32)		27 (24)	33 (27)	61 (31)	
High school	21 (33)	29 (30)	68 (33)		35 (30)	42 (34)	69 (35)	
Some college or college graduate	21 (33)	34 (35)	73 (35)		53 (46)	47 (39)	65 (33)	
Tobacco use N (%)				0.300				0.037
Never	12 (19)	27 (28)	45 (22)		33 (29)	26 (21)	69 (35)	
Former	37 (58)	44 (46)	95 (46)		41 (36)	51 (42)	53 (27)	
Current	15 (23)	25 (26)	67 (32)		41 (36)	45 (37)	73 (37)	
Alcohol drinks/week N (%)				0.013				0.438
None (0 drinks)	24 (38)	41 (43)	106 (51)		58 (50)	73 (60)	119 (61)	
Moderate (1–7 drinks)	18 (28)	37 (39)	71 (34)		42 (37)	36 (30)	57 (29)	
Excessive (>7 drinks)	22 (34)	17 (18)	30 (15)		15 (13)	12 (10)	19 (10)	
Drug use N (%)								
Marijuana	21 (33)	29 (31)	44 (21)	0.078	25 (22)	18 (15)	25 (13)	0.117
Stimulant	13 (20)	8 (8)	17 (8)	0.027	10 (9)	7 (6)	7 (4)	0.154
Other non-stimulant	6 (9)	6 (6)	10 (5)	0.368	1 (1)	2 (2)	3 (2)	1.000
Hepatitis C virus N (%)				0.709				<0.0001
Uninfected	53 (85)	86 (89)	170 (83)		88 (77)	82 (70)	164 (88)	
Cleared	2 (3)	3 (3)	8 (4)		3 (3)	13 (11)	8 (4)	
Chronic	7 (11)	7 (7)	26 (13)		23 (20)	22 (19)	15 (8)	
Co-Morbidities								
Clinically- relevant Depressive symptoms (CES-D≥16) N (%)	24 (38)	22 (23)	47 (23)	0.060	31 (27)	33 (27)	51 (26)	0.989
Hypertension N (%)	19 (30)	29 (30)	99 (48)	0.003	37 (32)	45 (37)	85 (44)	0.125
Diabetes (self- reported) N (%)	6 (9.4)	10 (10.4)	54 (26.1)	0.001	11 (9.6)	23 (18.9)	48 (24.6)	0.005
HIV specific factors								
CD4+ T-cell count, cells/µL								0.985
<200					4 (4)	3 (3)	6 (3)	
200–499					32 (28)	33 (27)	50 (26)	
>500					78 (68)	85 (70)	135 (71)	
Suppressed HIV-1 RNA					90 (79)	90 (75)	152 (80)	0.523
History of AIDS					38 (33)	49 (40)	74 (38)	0.506
Use of efavirenz					65 (56)	84 (69)	131 (67)	0.956

*Cross-sectional associations of BMI and WC with NP by neuropsychological domain at baseline*

Multivariable-adjusted associations of BMI and WC categories with NP by domain at baseline (**Table 2**) showed that obese WLWH performed more poorly on executive function (β= -2.17, 95%CI [-4.38, -0.04]) compared to WLWH who had a healthy BMI. In addition, WLWH who had a high-risk WC performed worse on motor function (β= -2.37, 95%CI [-4.26, -0.48]) No additional statistically significant associations were observed among WLWH or WWOH, despite several consistently negative β-coefficients, suggesting poorer performance associated with higher anthropometric adiposity measures.

**Table 2:** Baseline cross-sectional associations of domain-specific neuropsychological performance by overweight and obesity status among women without HIV (WWOH) and women living with HIV (WLWH).

Primary predictors	Neuropsychological domains					
	Memory β (95% CI)	Learning β (95% CI)	Attention/working memory β (95% CI)	Executive function β (95% CI)	Processing speed β (95% CI)	Motor function β (95% CI)
BMI						
WWOH						
Overweight (vs. healthy)	-2.58 (-5.64, 0.49)	-0.33 (-3.27, 2.61)	1.17 (-1.79, 4.13)	-1.46 (-4.27, 1.32)	-2.30 (-5.09, 0.48)	-0.04 (-2.90, 2.82)
Obese (vs. healthy)	-1.49 (-4.29, 1.30)	-0.65 (-3.33, 2.03)	1.62 (-1.08, 4.32)	-1.15 (-3.69, 1.40)	-1.41 (-3.94, 1.14)	1.12 (-1.49, 3.72)
Obese (vs. overweight)	1.08 (-1.30, 3.45)	-0.32 (-2.59, 1.96)	0.45 (-1.84, 2.75)	0.31 (-1.84, 2.48)	0.90 (-1.26, 3.06)	1.16 (-1.05, 3.37)
WLWH						
Overweight (vs. healthy)	-0.35 (-2.69, 1.98)	-0.61 (-2.86, 1.63)	-0.38 (-2.96, 2.20)	-1.32 (-3.73, 1.09)	-2.14 (-4.37, 0.10)	-1.58 (-3.82, 0.65)
Obese (vs. healthy)	0.30 (-1.84, 2.45)	1.01 (-1.05, 3.07)	-0.19 (-2.55, 2.18)	**-2.17 (-4.38, -0.04)**	-1.53 (-3.58, 0.52)	-1.47 (-3.52, 0.58)
Obese (vs. overweight)	0.66 (-1.43, 2.74)	1.73 (-0.27, 3.73)	0.19 (-2.10, 2.48)	-0.85 (-3.00, 1.30)	0.61 (-1.39, 2.60)	0.11 (-1.87, 2.11)
Waist circumference^b^						
WWOH						
High risk (vs. low risk)	-0.18 (-2.59, 2.30)	-0.01 (-2.28, 2.30)	0.74(-1.60, 3.09)	-0.91 (-3.12, 1.30)	-0.17 (-2.38, 2.04)	0.09 (-2.16, 2.34)
WLWH						
High risk (vs. low risk)	0.13 (-1.83, 2.08)	0.76 (-1.14, 2.66)	-0.11 (-2.30 2.07)	-1.38 (-3.45, 0.69)	-1.05 (-2.97, 0.86)	**-2.37 (-4.26,- 0.48)**

^a^Healthy BMI 20.00–24.99 kg/m^2^; overweight 25.00–29.99 kg/m^2^; obese ≥30.00 kg/m^2^.

^b^Higher WC ≥88 cm (≥35 inches).

All models adjusted for Center for Epidemiologic Studies Depression Scale score; use of tobacco, alcohol, marijuana, stimulants, or other drugs; hypertension and diabetes.

Models among WLWH also included baseline CD4 count, suppressed HIV-1 RNA, history of AIDS, use of efavirenz. Bold values means P<0.05.

*Baseline BMI and WC in association with repeated measures of NP by neuropsychological domain*

Longitudinally, most associations were observed among WWOH. WWOH who were overweight versus healthy BMI declined on memory (β= -2.67, 95%CI [-5.40, -0.07]). However, WWOH who were obese versus overweight based on BMI improved on memory (β=2.19, 95%CI [0.13, 4.26]), as did WWOH with an at-risk WC (β=1.81, 95%CI [0.19,3.44]).

In addition, in WWOH only, increasing continuous baseline WC, was associated with greater declines in executive function (β=-0.06, 95%CI [-0.11, -0.01]), processing speed (β=-0.05, 95%CI [-0.09, -0.01]), and motor (β=-0.04, 95%CI [=0.09, -0.00]) performance (**Table 3**). No other statistically significant associations were observed.

**Table 3:** Multivariable-adjusted associations of baseline body mass index (BMI) and waist circumference (WC) in association with longitudinal change in domain-specific neuropsychological performance among women without HIV (WWOH) and women living with HIV (WLWH).

Primary predictors	Neuropsychological domains					
	Memory β (95%CI)	Learning β (95%CI)	Attention/ working memory β (95%CI)	Executive function β (95%CI)	Processing speed β (95%CI)	Motor function β (95%CI)
Body Mass Index^a^						
WWOH						
Continuous BMI	-0.05 (-0.04, 0.15)	0.04 (-0.05, 0.13)	-0.03 (-0.07, 0.08)	-0.07 (-0.15, 0.10)	-0.07 (-0.14, 0.01)	-0.06 (-0.15, 0.03)
Overweight (vs. healthy)	-2.67 (-5.40, -0.07)	-0.59 (-3.16, 1.98)	0.34 (-1.85, 2.54)	-1.36 (-3.80, 1.06)	-1.71 (-4.17, 0.75)	-0.05 (-2.68, 2.57)
Obese (vs. healthy)	-0.47 (-2.87, 1.93)	1.00 (-1.21, 3.20)	1.90 (-0.03, 3.84)	-1.04 (-3.22, 1.14)	-1.04 (-3.12, 1.04)	0.28 (-2.30, 2.87)
Obese (vs. overweight)	2.19 (0.13, 4.26)	1.59 (-0.38, 3.57)	1.56 (-0.24, 3.36)	0.32 (-1.48, 2.12)	0.66 (-1.38, 2.71)	0.33 (-1.36, 2.02)
WLWH						
Continuous BMI	0.02 (-0.05, 0.09)	**0.07 (0.00, 0.15)**	-0.03 (-0.11, 0.05)	-0.06 (-0.13, 0.02)	0.01 (-0.06, 0.08)	-0.00 (-0.07, 0.07)
Overweight (vs. healthy)	-1.37 (-3.28, 0.54)	-1.63 (-3.53, 0.26)	-0.01 (-2.08, 2.05)	-0.71 (-2.89, 1.48)	-1.41 (-3.32, 0.51)	-1.39 (-3.49, 0.70)
Obese (vs. healthy)	0.532(-1.93, 1.28)	-0.24 (-1.87, 1.39)	0.25 (-1.69, 2.18)	-1.42 (-3.42, 0.57)	-0.79 (-2.62, 1.04)	-1.46 (-3.36, 0.44)
Obese (vs. overweight)	1.04 (-0.71, 2.80)	1.39 (0.34, 3.11)	0.26 (-1.74, 2.60)	-0.76 (-2.56, 1.11)	0.61 (-1.13, 2.36)	-0.07 (-2.04, 1.91)
Waist circumference^b^						
WWOH						
Continuous WC	0.01 (-0.03, 0.06)	0.01(-0.03, 0.06)	-0.02 (-0.07, 0.02)	**-0.06 (-0.11, -0.01)**	-0.05 (-0.09, -0.01) ^*^	**-0.04 (-0.09, -0.00)**
Higher (vs. lower)	1.81 (0.19, 3.44)	1.21 (-0.49, 2.90)	0.85 (-0.64, 2.83)	-0.51 (-1.79, 0.77)	0.42 (-0.92, 1.78)	-0.08 (-1.32, 1.48)
WLWH						
Continuous WC	0.01 (-0.05, 0.03)	0.02 (-0.02, 0.06)	-0.04 (-0.08, 0.00)	-0.02 (-0.07, -0.02)	-0.00 (-0.04, -0.04)	-0.01 (-0.05, -0.03)
Higher (vs. lower)	-0.13(-1.47, 1.20)	0.26 (-1.11, 1.64)	-0.09 (-1.25, 1.43)	-0.90 (-1.96, 0.15)	0.07 (-1.12, 0.98)	-0.90 (-1.98, 1.18)

^a^Healthy BMI 20.00–24.99 kg/m^2^; overweight 25.00–29.99 kg/m^2^; obese ≥30.00 kg/m^2^.

^b^Higher WC ≥88 cm (≥35 inches).

All models adjusted for Center for Epidemiologic Studies Depression Scale score; use of tobacco, alcohol, marijuana, stimulants, or other drugs; hypertension and diabetes.

Models among WLWH also included baseline CD4 count, suppressed HIV-1 RNA, history of AIDS, use of efavirenz. Bold values means P<0.05.

## Discussion

Among WLWH and WWOH participating in the WIHS, we used a similar study design and analysis strategy that was published among MLWH and MWOH participating in the MACS (27). Overall associations between anthropometric measures of adiposity and NP decline were not as strong and less consistent among the women compared to the men. Among WLWH, we observed cross-sectional associations between total and central obesity with poorer executive and motor function, respectively. Poorer motor function was also observed among MWOH with higher BMI and at-risk WC among MLWH and MWOH. Longitudinally, the associations between baseline higher total adiposity and NP were mixed for memory among WWOH, however increasing WC was associated with decreasing executive, processing speed and motor function, which is somewhat consistent with the MACS analysis among MWOH (27). Contrastingly, an obese, at-risk waist was associated with improving memory performance over time among WWOH.

Despite similar longitudinal cohort studies of women and men with and without HIV infection, lack of similar results between the WIHS and the MACS cohorts could be due to sample heterogeneity including differences in biological sex; median baseline age (despite the age inclusion criterion of ≥40 years for analyses in both women and men, men were on average, older (50 years) than women (45 years)); baseline BMI (men had a lower prevalence (29%) of overweight and obesity versus 39% in women); race (men were predominantly White (69.5%) versus 10% of women); and men had higher socioeconomic status and educational attainment.

Other analyses among WIHS participants at different stages of the adult life course have suggested mixed associations between anthropometric measures of overweight and obesity (i.e., BMI, WC) and NP. Cross-sectionally, based on NP data collected from 2005-2008, among WLWH who were average age 38 years, an underweight BMI (<18.5 kg/m^2^) was associated with poorer NP as evidenced by poorer executive function (longer Trails A and Trails B completion) and poorer processing speed (longer test completion times). In contrast, women with an obese BMI (≥30 kg/m^2^) was related to better executive function (via Trails B) and also worse executive performance (via the Stroop interference test). Among obese WWOH, worse performance on the Stroop color naming test, a measure of processing speed, was observed.

We also analyzed 10-year change in BMI (from 2009-2019) in association with subsequent NP among a subset of WIHS participants who transitioned from average age 40 to 50 years in Brooklyn, New York and Chicago, Illinois, USA. Increasing BMI over a 10-year follow-up predicted slower processing speed (P=0.043) among all women; and among WLWH, poorer EF (P=0.01) and global (P=0.04) performance. Therefore, among WLWH, over subsequent 10 years follow-up with receipt of increasingly effective ART, expected trajectories of increasing aging-associated anthropometric and metabolic measures of adipose tissue influenced associations among BMI, WC, and NP. These associations differed in WWOH.

Important issues related to analyses across HIV aging studies of vascular risk factors and NP require additional exploration. First, the BMI and WC cut-offs that are traditionally used to classify people as overweight and obese were defined for adult non-Latinx White and WWOH populations (42–45). While data suggest the validity of these cut-offs among non-White populations for predicting cardiovascular disease, diabetes, and mortality, other cut-offs may be more relevant to NP and brain aging, especially for underrepresented Black WLWH (46, 47). In 2004, the World Health Organization Expert Panel (48) put forward pragmatic rather than evidence-based BMI cut-offs specific for Asian people (healthy BMI, 18.5–23.9 kg/m^2^; overweight, 24.0–27.9 kg/m^2^; obese ≥28.0 kg/m^2^) (49). However, no guidance has been published for Non-Latinx Black people, people of African descent, or other ethnic and racial people groups. This was the rationale for using both continuous and traditional categorical anthropometric exposure variables in our analysis. Second, overweight and obesity cut-offs for older adults are debated (50–52). Studies suggest that a higher cut-off for overweight and obesity among older adults are more appropriate (50). Third, chronological age and temporality in adiposity-NP associations over the life course must continue to be explored (53). Here we evaluated adiposity and NP among women who were age ≥40 years at baseline (median age, 45 years) over approximately 8 years. Median age 45 years is approximately 20 years before a potential late-onset AD diagnosis, and perhaps too early in the aging brain process to observe more apparent and/or consistent cross-sectional and longitudinal associations of vascular risk factors including adiposity with NP. Fourth, related to historical and ongoing observational studies of older adults, birth cohort differences are important. Being born in the mid-20^th^ century compared to earlier years will be accompanied by global secular trends such as higher population levels of BMI and educational attainment. Fifth, adverse social determinants of health such as early-life stressors, trauma, and barriers to healthcare services (31, 54, 55), common among WIHS participants and other WLWH, are associated with overweight and obesity, poorer NP, and late-life AD and VCID outcomes in uninfected populations (56, 57). Sixth, mid-life fluctuations in NP are more likely to occur with co-morbidities (58), and misuse of alcohol and other substances (59). However, we adjusted for these factors at baseline. Seventh, we hypothesized that ART may have a neuroprotective role since associations of higher adiposity with declining NP were accentuated in WWOH. This potential protective effect of ART is supported by ongoing clinical trials exploring the use of efavirenz, nucleoside reverse transcriptase inhibitors, and nucleoside analogs in the treatment of AD without HIV [49]. Finally, more research generally, is needed on the role of endocrine adipose tissue and the fat-brain axis.

This study has several strengths. The WIHS was a large longitudinal cohort study with repeated NP assessments in well-characterized WLWH and WWOH who are underrepresented in the brain health literature, based on HIV status, Non-Latinx Black race and health disparities. Easily obtained anthropometric measures of obesity (BMI and WC) and NP were rigorously collected over time using standardized protocols. Trained research study staff measured body weight, body height, and WC, and conducted face-to-face NP assessments. WLWH were well-phenotyped and had to be taking ≥2 antiretroviral therapies and demonstrate an HIV-1 RNA <400 copies/mL at more than 80 percent of their visits. Finally, our statistical approaches were appropriate for the study design and data available. For our descriptive **Table 1**, we used the Wilcoxon rank sum test to address a few small cell sizes. In contrast, the NP outcomes are continuous and measured in hundreds of women for which linear regression models and repeated measures analyses with generalized estimating equations work well because of large sample theory, even if the T-score distributions differ somewhat from assessment to assessment.

Limitations of our study should also be considered. Inclusion of women ≥40 years at baseline with the eldest age 56 years, may have minimized robust associations that are more commonly observed among older adults. Our cohort did not include brain or whole-body composition fluid or imaging biomarker data. We did not adjust for multiple comparisons given the small sample size, however we provided β-coefficients and 95%CI for each comparison. Information on other neurological outcomes that are associated with HIV, e.g., peripheral neuropathy, were not adequately collected in the WIHS. The clinical relevance of our findings given the observed point estimates and 95%CIs, may be limited during this chronological stage of a woman’s life course. While a strength, our inclusion criteria for WLWH of HIV-1 RNA <400 copies/mL and ≥2 ART may have reduced variability in adiposity exposures and NP outcomes since some ART may be beneficial for NP and/or cause weight gain. In addition, as research study participants who receive regular information about their overall health, these women differ from WLWH and WWOH who do not participate in research studies and/or do not receive adequate healthcare. However continued monitoring of BMI and WC trajectories into later life will provide important insights regarding associations observed in specific chronological age observation windows. In addition, inclusion of women based on completion of only two (or more) NP assessments did not allow for a true analytic trajectories approach over time among all participants. Finally, our duration of follow-up was slightly less than 10 years, which may have been too short at this adult life stage. Longer follow-up to older ages and additional measures of NP will clarify trajectories and allow understanding of the temporal nuances of this association.

## Data availability statement

Publicly available datasets were analyzed in this study. This data can be found here: https://airtable.com/shrVDP51W5J2qcNeT. The WIHS provides deidentified data for replication studies andother forms of analytic inquiry on approval by the studyinvestigators (https://statepi.jhsph.edu/mwccs/).

## Ethics statement

The studies involving human participants were reviewed and approved by Multiple sites IRBs: The study was approved by the Institutional Review Board (IRB) of each participating site in accordance with the Declaration of Helsinki. Each participating institution’s IRB has been formally designated to review and monitor biomedical research involving human subjects, with the primary responsibility being the protection of subjects from undue risk and from deprivation of personal rights and dignity, which are the cornerstones of ethical research (For the southern sites: University of Mississippi Medical Center; University of North Carolina at Chapel Hill; University of Alabama at Birmingham; University of Miami and Emory University. Brooklyn: SUNY Downstate Medical Center and Kings County Medical Center. Bronx: Montefiore Medical Center; Beth Israel Medical Center; Mount Sinai School of Medicine. Chicago: Cook County Health & Hospitals System; Rush University Medical Center; University of Illinois at Chicago. San Francisco: University of California, San Francisco; Alameda Health System; Sutter Health; Santa Clara Valley Medical Center; San Mateo Medical Center. District of Columbia: Georgetown University; Montgomery County Department of Health and Human Services; Inova; Howard University and Whitman-Walker Clinic. The patients/participants provided their written informed consent to participate in this study. The institutional review boards of each WIHS clinical researchsite approved the WIHS research protocol and all participantsprovided written informed consent.

## Author contributions

DRG and LHR: Major role in the acquisition of data, study concept or design, analysis or interpretation of data, drafting/revision of the manuscript. MK: contributed to the design of the study, the statistical analysis, interpretation of the study findings and drafting the manuscript. AAApp contributed to interpretation of the study findings and drafting the manuscript. AAAdi: major role in the acquisition of data. SH and HM: major role in the acquisition of the data; study concept or design, contributed to interpretation of the study findings and drafting the manuscript. MF, ED, WM, CM, MP, AS, PT and KW: major role in the acquisition of data; contributed to interpretation of the study findings and drafting the manuscript. All authors contributed to the article and approved the submitted version.
